# Association of a Simulated Institutional Gender Equity Initiative With Gender-Based Disparities in Medical School Faculty Salaries and Promotions

**DOI:** 10.1001/jamanetworkopen.2018.6054

**Published:** 2018-12-21

**Authors:** Avani D. Rao, Sarah E. Nicholas, Bartlomiej Kachniarz, Chen Hu, Kristin J. Redmond, Curtiland Deville, Jean L. Wright, Brandi R. Page, Stephanie Terezakis, Akila N. Viswanathan, Theodore L. DeWeese, Barbara A. Fivush, Sara R. Alcorn

**Affiliations:** 1 Department of Radiation and Molecular Radiation Sciences, Johns Hopkins University School of Medicine, Baltimore, Maryland; 2 Department of Plastic and Reconstructive Surgery, Johns Hopkins University School of Medicine, Baltimore, Maryland; 3 Department of Oncology—Biostatistics and Bioinformatics, Johns Hopkins University School of Medicine, Baltimore, Maryland; 4 Department of Pediatrics, Johns Hopkins University School of Medicine, Baltimore, Maryland

## Abstract

**Importance:**

Despite progress in narrowing gender-based salary gaps, notable disparities persist in the scientific community. The significance of pay difference may be underestimated, with little data evaluating its effect on lifetime wealth after accounting for factors like time to promotion and savings.

**Objectives:**

To characterize gender disparities in salary and assess the outcomes associated with a gender equity initiative (GEI).

**Design, Setting, and Participants:**

Quality improvement study with simulations of salary and additional accumulated wealth (AAW) using retrospectively reviewed Johns Hopkins University School of Medicine annual salary and promotion data. All academic faculty were included in the faculty salary analysis from 2005 (n = 1481) and 2016 (n = 1885).

**Main Outcomes and Measures:**

Salary and longitudinal promotion data from 2005 to 2016 were used to estimate gender-based differences in salary and time to promotion. The effect of these differences on total salary and AAW, including retirement and salary-based investments, was simulated for a representative male and female faculty member over a 30-year career in 3 scenarios: (1) pre-GEI, (2) post-GEI, and (3) in real time for GEI, beginning with and progressing through these initiatives.

**Results:**

Analyses of salaries of 1481 faculty (432 women) in 2005 and 1885 faculty (742 women) in 2016 revealed that a decade after GEI implementation, the overall mean (SE) salary gap by gender decreased from −2.6% (1.2%) (95% CI, −5.6% to −0.3%) to −1.9% (1.1%) (95% CI, −4.1% to 0.3%). Simulation of pre-GEI disparities correlated with male faculty collecting an average lifetime AAW of $501 416 more than the equivalent woman, with disparities persisting past retirement. The AAW gap decreased to $210 829 in the real-time GEI simulation and to $66 104 using post-GEI conditions, reflecting success of GEI efforts.

**Conclusions and Relevance:**

Even small gender-based salary gaps are associated with substantial differences in lifetime wealth, but an institutional commitment to achieving equitable promotion and compensation for women can appreciably reduce these disparities. The findings of this study support broad implementation of similar initiatives without delay, as results may take more than a decade to emerge. A modifiable version of the simulation is provided so that external users may assess the potential disparities present within their own institutions.

## Key Points

**Question:**

What is the true magnitude of differences in salary, time to promotion, and accumulated wealth between male and female faculty, and how are these measures associated with a set of institutional gender equity initiatives?

**Findings:**

In this quality improvement simulation study of salaries of 1481 faculty, the salary gap and time to promotion decreased after implementation of gender equity initiatives; however, small persistent differences were still associated with substantial disparities.

**Meaning:**

Residual gender-based salary gaps may lead to substantial differences in accumulated wealth over the career course and into retirement, but institution-wide promotion of equity initiatives can slowly begin to narrow the disparity.

## Teaser

This quality improvement study uses a simulation to project differences in salary, time to promotion, and accumulated wealth between male and female medical school faculty before and after the implementation of an institutional gender equity initiative.

## Introduction

Although antidiscrimination laws and policies have improved the position of women over time, gender-based compensation disparities for women in science have persisted.^1,2,3,4^ From the available reports, compensation for men exceeds that for women by approximately 7% to 8% among US physician researchers,^2,3^ nearly 20% among Japanese surgeons,^5^ and 25% to 40% between scientists in public sectors in Europe^6,7^ even after controlling for other factors such as years of experience and rank.

With increasing attention on such inequities faced by female scientists, the Johns Hopkins University School of Medicine (JHUSOM) reviewed institutional data and noted that there was a lag in promotion of women to full professorship despite the presence of a significant proportion of women at the lower faculty ranks. In response, the JHUSOM created the Committee on Faculty Development and Gender in 2002. The committee first investigated barriers to career promotion by (1) reviewing faculty representation, attrition, and promotion rates for women; (2) conducting faculty surveys and departmental director interviews to identify sources of differences in career progression; and (3) performing annual faculty salary analyses (FSAs) to track salary inequities. The committee then offered specific recommendations, summarized in the Box.

Box. **Summary of Recommendations From the 2005 Final Report of the Committee on Faculty Development and Gender From the Johns Hopkins University School of Medicine**

**Key Recommendations**

Elevate the goal of gender equity to an essential mission of the institution
Achieve salary equity through continued close review of faculty salary analyses, with leadership directing actions toward departments with salary disparities in an effort to narrow the gap
Discourage meetings and conferences from being scheduled outside of normal business hours to promote inclusion of women
Dedicate financial resources toward the recruitment and retention of women to senior faculty ranks
Expand sexual harassment educational curriculum
Interview departing women faculty to identify root causes for attrition
Create a standing oversight committee to monitor issues related to gender equity
Conduct a faculty survey in 3 years to assess progress

An oversight committee was created in 2006 to supervise implementation of these recommendations. In particular, leadership used annual FSAs to target remediation actions toward departments with higher salary gaps. Committees created templates that standardized criteria for promotion across departments. Financial and human resources were allotted for the recruitment and retention of women faculty. The Office of Women in Science and Medicine was created to increase pathways to leadership, and more than 600 women have participated in its programming to date. To increase transparency of efforts, JHUSOM continues to publish annual FSAs and interval reports on the status of women.

Collectively, these efforts—referred to as gender equity initiatives (GEI)—have narrowed salary and promotional gaps at the JHUSOM. However, a small overall gender-based salary difference of 1.9% persists. While our institutional goal is to achieve 0% difference by the 2018 fiscal year, others may argue that such single-digit residual gaps are relatively insignificant, thus negating the need for additional intensive measures to eliminate them completely. Yet when accounting for the cumulative effect of salary inequity on retirement and investment savings, compounded with issues such as increased time to promotion (TTP) and longer life expectancy for women, the total impact of even small gaps may have profound implications for lifetime earning potential and retirement for women. To better illustrate the true costs of salary inequities, we have simulated the total disparity in lifetime wealth created by residual gender-based salary differences over the course of a career and into retirement. In doing so, we highlight the impact of more than a decade of work at JHUSOM and offer our GEI efforts as a potential framework for use across institutions with the goal of eliminating this gap.

## Methods

### Source Data

Annual FSAs were conducted for all JHUSOM faculty members for fiscal years 2005 to 2016, including information on department, self-reported gender, degree, rank, years in rank, and fiscal year salary. Data for both MD and non-MD faculty were considered in our evaluation. For the purposes of our analysis, annual salary for a given fiscal year included full-time equivalent base salary and supplemental salary for administrative, educational, or clinical roles. Bonus salary data were not available. All salary values were reported in US dollars. This study met the criteria for institutional review board review waiver. The authors followed Standards for Quality Improvement Reporting Excellence (SQUIRE) 2.0 reporting guidelines for reporting quality improvement.

Deidentified information from annual FSAs was analyzed by the Johns Hopkins Bloomberg School of Public Health and published internally to the JHUSOM community. Each FSA reported unadjusted mean annual salary by gender, subcategorized by faculty rank (assistant professor, associate professor, or professor) and degree (MD vs non-MD). Annual FSA reports also estimated gender-based difference in mean annual salary using multivariable linear regressions. These regressions applied a logarithmic transformation to salary to minimize the impact of outliers and adjusted for gender, department, department-specific rank, degree, and years in rank. As per model specifications, the resultant regression coefficients for gender corresponded to the estimated percentage difference in salary comparing women with otherwise similar men, with negative values indicating women earning less than men. Regression models available in the FSA included the overall gender difference and the rank-specific gender difference in salary.

Median TTP by gender was obtained from registrar records for 2 cohorts of faculty hired at the rank of assistant professor or associate professor. The pre-GEI era cohort reflects TTP for faculty hired between 1989 and 1990 (prior to GEI implementation) and was examined in November 2005. The GEI era cohort was hired between 2005 and 2017 and was examined in January 2018.

### Outcome Measures and Simulated Scenarios

Simulations were performed to model the association of gender-based differences in salary and TTP with estimated annual salary and additional accumulated wealth (AAW) over the course of a 30-year career for otherwise identical representative male and female full-time faculty members. In the simulation, the faculty was hired as an assistant professor and promoted to associate professor and then professor. Corresponding salary and TTP were estimated using gender- and rank-specific mean and median values for these variables from annual FSA and registrar cohort data, respectively. To demonstrate the impact of institutional efforts to decrease the compensation gap on AAW, we simulated 3 scenarios:

1. Pre-GEI: Gender-based differences in salary over the career course were estimated using the 2005 fiscal year FSA. Gender differences in TTP at each promotion were obtained using pre-GEI era cohort data. As such, this scenario simulates gender differences for lifetime AAW for a faculty member hired in pre-GEI conditions and for whom the pre-GEI salary and TTP inequities remained constant throughout the career course.
2. Post-GEI: Gender differences in salary over the career course were estimated using the 2016 fiscal year FSA. Gender differences in TTP were obtained using GEI era cohort data. As such, this scenario reflects the conditions faced by a faculty member hired in post-GEI conditions and thus subject to narrowed salary and TTP gaps throughout the career course.
3. Real-time GEI: Data from the 2005 fiscal year FSA were used to simulate baseline gender differences in salary for an assistant professor hired at that time. Gender differences in TTP were estimated using GEI era cohort data. At the time of promotion from assistant professor to associate professor (in *x* years for the man and *y* years for the woman) and from associate professor to professor (in *x′* years for the man and *y′* years for the woman), an increase in the gender-specific salary reflecting values reported in the corresponding year’s FSA were applied as follows: baseline salary + (*x, x′, y, y′*). Thus, this scenario simulates the potential impact of salary and promotional differences for a faculty member hired in pre-GEI conditions but subject to the real-time efforts of the GEI.

To assess the differential effects of gender inequities by promotional rank, a secondary set of simulations were applied to each of the 3 scenarios described. In these simulations, the representative faculty members’ careers took the following paths: (1) no promotion beyond assistant professor, (2) promotion only to associate professor, or (3) promotion through full professor (equivalent to the assumptions of the scenarios described).

For each scenario, we created a projection of both annual salary and AAW based on gender differences in salaries and TTP over the course of a 30-year career.

### Annual Salary

In all 3 scenarios, the starting salary of the representative male assistant professor was set at $108 750, which is the average of the mean salaries for a male MD and male non-MD assistant professor from the 2005 FSA. To ensure that comparisons were made between otherwise similar men and women, we calculated the corresponding female assistant professor’s salary using the rank-specific gender difference in salary for a given scenario, as estimated by the FSA’s adjusted linear regression model. This approach was again repeated at each appropriate time for promotion to associate professor and to professor. Simulations of salary before and after the GEI assumed a 3% annual increase for cost of living for each year at a given rank until promotion.^8^

### Additional Accumulated Wealth 

Additional accumulated wealth included estimated retirement savings and other salary-based investments accrued over time. A standard employer contribution to the pretax retirement account was set at 12% of gross income, reflecting our institutional practice. Each simulation included a pretax retirement account deposited with maximal employee contributions throughout the 30-year career. Initial maximum contributions were indexed to an inflation rate of 2%^9^ and set at $18 000 through age 50 as per US federal regulations, with an additional 33% catch-up contribution after age 50. Take-home pay was estimated as monies received from salary after employee pretax retirement contributions and deduction of marginal tax rate of 33.45% (8% local, 24% US federal income, and 1.45% Medicare). The simulation assumed equivalent standard of living—and thus equivalent annual expenses—for both genders. For the simulation, we set the annual expenses equal to the lowest take-home pay between the genders. Any take-home pay in excess of annual expenses for a given gender was assumed to be invested in a long-term diversified portfolio.

The equity and bond composition used for retirement and salary-based investments is described in the eFigure in the Supplement. Expected rate of investment returns were 7% for equity markets and 3% for bond markets, based on an assumed risk-free rate of 2%, equity risk premium of 5%, and bond risk premium of 1%.^10,11^

### Postretirement Wealth

Using the real-time GEI scenario, we calculated postretirement AAW and income, starting with total AAW accrued at the time of simulated retirement by gender. Additional contribution of retirement savings to gross postretirement income was calculated based on a graduated annuity with a 20/80 equity/bond investment portfolio return and 2% inflation-adjusted income growth throughout retirement. We used US Social Security Life Tables^12^ to estimate life expectancy and anticipate years in retirement by gender. Differences in postretirement wealth available for use over the course of remaining life were compared between genders.

### Statistical Considerations

In addition to reporting outcomes for the JHUSOM, a goal of our study was to create an editable version of the simulation that external users could modify to reflect conditions faced at their own institutions. In light of this aim, our methodology was selected to optimize generalizability and use types of data most likely to be accessible. However, our ability to accurately estimate measures of variance or perform tests of significance for our own institutional data was compromised in order to meet this greater goal of generalizability.

For example, to protect faculty privacy, the annual FSA information analyzed at JHUSOM is not published at an individual level; instead, it is best considered as repeated cross-sectional data. We anticipated this to be the most likely level of available data for external users as well. However, the deidentified nature of the data prohibited us from direct longitudinal analysis of career trajectories for specific faculty over time. Moreover, subgroups used to report aggregate measures of central tendency, regression coefficients, and variance were not uniform within or across FSA reports. To enhance comparability, some subgroups (such as MD and non-MD faculty) were combined. Given these limitations, we determined that we could not accurately specify the large number of assumptions needed in order to capture variance and perform hypothesis testing for outcomes like AAW by gender. Microsimulation methods were considered but ultimately not used because the detailed procedures required to calibrate such simulations to our population would potentially compromise applicability to external users.

As per FSA analyses, the adjusted regression coefficients and standard errors reflecting the estimated overall and rank-specific gender difference in salary were reported, and 95% confidence intervals were specified.

Simulation projections were developed and displayed using Microsoft Excel 2016 and Shiny version 1.0.5 (RStudio).

## Results

A total of 1481 faculty (432 women) in 2005 and 1885 faculty (742 women) in 2016 completed the FSA. Table 1 shows participant characteristics from the pre- and post-GEI cohorts. In 2005, 31% of full-time faculty were women, as compared with 39% in 2016. In an adjusted analysis, women’s salaries were estimated to be a mean (SE) of −2.6% (1.2%) (95% CI, −5.6% to −0.3%) lower than men’s salaries in 2005 across rank, with the overall gap narrowing to −1.9% (1.1%) (95% CI, −4.1% to 0.3%) in 2016. Salary differences generally narrowed with increasing academic rank.

**Table 1.** Descriptive Statistics of Faculty Cohorts Included in Pre-GEI and Post-GEI and Variables Used in Each of the 3 Simulation Scenarios

Characteristic	Pre-GEI	Post-GEI	Real-time
Starting year FSA data	2005	2016	NA
FSA faculty demographic characteristics, No. (%)			
Total faculty	1481	1885	NA
Women	452 (31)	742 (39)	NA
Men	1029 (69)	1143 (61)	NA
Assistant professor, No. (%)	705	897	NA
Women	274 (39)	443 (49)	NA
Men	431 (61)	454 (51)	NA
Associate professor, No. (%)	390	509	NA
Women	108 (28)	195 (38)	NA
Men	282 (72)	314 (62)	NA
Professor, No. (%)	386	489	NA
Women	70 (18)	114 (23)	NA
Men	316 (82)	375 (77)	NA
Assistant professor annual salary for men, $	108 750^a^	108 750^a^	108 750^a^
Adjusted mean (SE) difference in salary between female vs male assistant professor [95% CI], %^b^	−2.90 (1.6) [−6.0 to 0.2]^a^	−3.40 (1.5) [−6.3 to −0.4]^c^	−2.90 (1.6) [−6.0 to 0.2]^a^
Time to promotion from assistant to associate professor, y			
Women	6	4.5	4.5
Men	5	4.9	4.9
Associate professor annual salary for men, $	159 342^d^	154 701^d^	156 350^e^
Adjusted mean (SE) difference in salary between female vs male associate professor [95% CI], %^b^	−3.60 (2.5) [−8.5 to 1.3]^a^	−0.60 (2.1) [−4.6 to 3.6]^c^	−3.70 (2.3) [−8.2 to 0.7]^e^
Time to promotion from associate to professor, y			
Women	7.8	5.3	5.3
Men	6.3	4.4	4.4
Professor annual salary for men, $	239 749^d^	225 987^d^	217 450^f^
Adjusted mean (SE) difference in salary between female vs male professor [95% CI], %^b^	−1.50 (2.8) [−7.0 to 4.0]^a^	0.10 (2.5) [−4.7 to 5.2]^c^	−1.5 (2.5) [−6.2 to 3.4]^f^

Abbreviations: FSA, faculty salary analysis; GEI, gender equity initiatives; NA, not applicable.

^a^ Data from the 2005 FSA.

^b^ Negative values indicate that women earned less than men.

^c^ Data from the 2016 FSA.

^d^ The 2005 FSA mean salary for men at a designated rank, adjusted for a 3% annual increase to account for cost of living per year corresponding to time to male promotion.

^e^ Data from 2009 FSA.

^f^ Data from the 2014 FSA.

### Simulated Association of Gender With Wealth in 3 Scenarios

**Figure 1.**
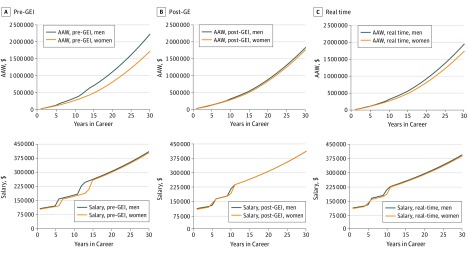
Simulations of the Association of Gender-Based Inequities With Compensation and Promotion From Assistant to Full Professor Simulations reflect pre–gender equity initiatives (GEI) (A), post-GEI (B), and real-time (C) scenarios. Whereas gender differences in annual salary are small across time in all 3 scenarios, the cumulative effect of salary and promotion disparities results in a significant difference in additional accumulated wealth (AAW) in the pre-GEI and real-time scenarios of $501 416 and $210 829, respectively. The AAW narrows to $66 104 using post-GEI conditions, reflecting success of GEI efforts.

Figure 1 demonstrates annual salary and AAW over the course of a 30-year career for an equivalent newly hired woman vs man in the pre-GEI, post-GEI, and real-time GEI scenarios. Salary and TTP data used for each scenario are outlined in Table 1.

Pre-GEI conditions (Figure 1A) resulted in an additional $501 416 AAW collected by the male faculty. This was composed of $35 000 from growth of employer retirement contributions and $466 415 from salary-based investments. Post-GEI (Figure 1B), the male faculty collected an additional $66 104 AAW, composed of $4831 from growth of employer retirement contributions and $61 273 from salary-based investments. Narrowed gaps in both TTP and compensation at associate professor and professor ranks in the post-GEI scenario corresponded to a reduction in the pre-GEI gender difference in AAW by 87%.

Real-time GEI simulation (Figure 1C) revealed the AAW gap was reduced to $210 829, composed of $15 883 from growth of employer retirement contributions and $194 946 from salary-based investments. The real-time GEI scenario was associated with a reduced pre-GEI gender difference in AAW by 58%.

### Secondary Simulations for Differential Association by Rank

**Figure 2.**
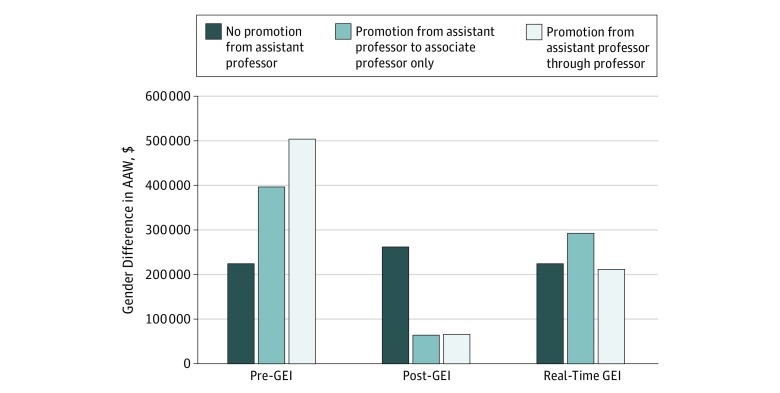
Simulations of the Association of Gender-Based Inequities With Compensation and Promotion in Different Career Paths Variations of pre–gender equity initiatives (GEI), post-GEI, and real-time scenarios to simulate the difference in additional accumulated wealth (AAW) by gender that may be experienced had both the male and female faculty members taken the following 30-year career paths: (1) no promotion beyond assistant professor, (2) promotion only to associate professor, or as simulated in the manner described in the text, (3) progressing through the ranks to full professor. The magnitude of differences in AAW is smallest in the post-GEI scenario due to significant narrowing of gender-based salary and promotional gaps. Residual AAW differences are most pronounced when the male and female faculty are not promoted beyond assistant professor due to baseline salary gaps of 2.9% to 3.4% across scenarios.

Figure 2 demonstrates secondary simulations performed to assess for differential effects of gender inequities by academic rank. Differences in AAW were consistent across all 3 scenarios for faculty remaining at the assistant professor rank throughout their careers. This is attributable to a starting salary gap of 2.9% to 3.4% across scenarios for this rank. Post-GEI gender differences in AAW were minimal for faculty promoted to associate professor or professor. For the real-time GEI scenario, persistent salary gaps correlated with larger gender differences in AAW compared with those in the post-GEI era despite similarly narrowed TTP gaps.

### Association of Life Span With Retirement Income

**Figure 3.**
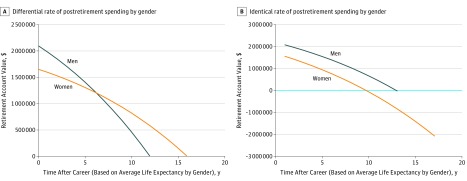
Simulated Postretirement Wealth and Income by Gender In this simulation of postretirement wealth and income by gender over the course of retirement years for the representative woman as compared with her male counterpart in the pre–gender equity initiatives (GEI), time from retirement to death was 17.25 years for the woman and 13.22 years for the man. Panel A shows the rate of spending required for each gender to use all postretirement wealth and income prior to death. Owing to the cumulative effect of salary and promotional disparities over her career course, a female faculty would be required spend her retirement savings at a rate 40% slower than her male counterpart to compensate for less retirement savings and longer life expectancy. Panel B demonstrates both genders spending postretirement wealth and income at an identical rate (set to the rate used by male faculty in A). Were she to spend her retirement wealth at the same rate as her male counterpart, her resources would be depleted 7 years prior to her death (represented by deficit of funds below the zero line).

Life expectancy in the United States for a woman now aged 35 years is 82.25 years, as compared with 78.22 years for a man aged 35 years. Thus, a woman can expect to spend 17.25 years in retirement past age 65 years as compared with 13.22 years for her male counterpart. To account for lower AAW and longer life expectancy, a woman would have to spend $0.60 for every $1 spent by her male counterpart in order for her resources to last through retirement (Figure 3A) in the pre-GEI scenario. For the real-time and post-GEI scenarios, this corresponds to women spending $0.68 to $0.73, respectively, for every $1 spent by their male counterparts. Were a woman to spend her retirement funds at the same rate as her male counterpart, the woman would be expected to deplete her account 7 years before her death in the pre-GEI scenario (Figure 3B) and 5 years and 4 years before her death in the real-time and post-GEI scenarios, respectively.

### Modifiable Simulation for External Users

To encourage external users to assess the effect of potential inequities faced at their own institutions, a modifiable version of our simulation can be found at https://dayflowerio.shinyapps.io/sra-gender-gap-r-shiny/. Default values entered reflect assumptions made for the pre-GEI scenario. Users can adjust these values to investigate the impact of assumptions made within our simulation.

## Discussion

Our simulations demonstrate that even single-digit gender differences in salaries are associated with substantial inequities in accumulated lifetime wealth over the course of an academic career in medicine, particularly when accounting for differences in promotional trajectory. In our pre-GEI simulation, this was related to a difference of more than half a million dollars in AAW. Moreover, these differences may track into retirement, where a woman would be required to spend her retirement savings at a rate 40% slower than her male counterpart to compensate for less retirement savings and longer life expectancy. To our knowledge, this is the first publication to describe the cumulative, nonlinear association of gender differences in salary and TTP with lifetime wealth for women in science.

Importantly, our analyses display the positive impact of an institutional commitment to eliminating gender inequities. By thoroughly investigating barriers faced by women faculty, the JHUSOM achieved an overall reduction of the salary gap from 2.6% to 1.9% and near equity in TTP between genders over the course of a decade. In our simulations, this was linked to a 7.6-fold reduction in the AAW gap as compared with pre-GEI conditions. Moreover, these data highlight that outcomes of directed change are not seen immediately; therefore, institutions without GEI in place should implement similar efforts promptly to prevent further delays in progress toward gender equity.

While much of the discussion to date has focused on gender-based salary gaps, our data emphasize the importance of TTP. In post-GEI conditions, the overall salary disparity of 1.9% was largely driven by persistent statistically significant gaps faced by women in the assistant professor rank, with salary differentials narrowing to near parity for associate professors and professors. Because GEI efforts also reduced TTP differences, the effect of early salary inequities on AAW was minimized for those promoted to higher ranks. Yet as Figure 2 demonstrates, AAW gaps remained substantial for women who were not promoted. Because women hold less than one-half and one-third of associate professor and professor positions in the United States, respectively,^13,14^ these data underscore the need to prioritize career development and supportive programs to encourage retention and promotion of qualified women to senior positions.

Additional steps may be required to further improve gender equity in science. Foremost, institutions must actively encourage women to enter and remain in the field. A recent survey showed that 35% of academic medical institutions did not have programs in place to support recruitment, promotion, and retention of women.^15^ Training in negotiation skills,^16^ education about implicit bias,^17,18^ coaching and mentoring, and financial support for professional development programs may further promote a supportive environment for women.^13^ Additionally, institutions must commit to integrating women into leadership roles.

### Limitations

A potential limitation of our study is that the nature of our source data limited our ability to perform tests of significance for outcomes of interest such as AAW differences by gender. As noted, we selected our methodology to optimize generalizability for external users, including through the use of our modifiable simulation platform. In turn, this platform enables users to evaluate the effects of their own institutional differences in salary by gender or other demographic characteristics of interest.

A second limitation is that we performed analyses with adjusted A+B data, as base salary (part A) was not reported separately. This could confound gender-based differences in salary if women are less likely to participate in or be compensated for administrative, educational, or clinical roles (part B). Moreover, we did not include bonus salary (part C) in our assessment. To address this, data on each separate component of salary will be acquired individually in future FSAs.

Our salary data were also averaged across MD vs non-MD faculty and adjusted for department. Because faculty who are not MDs or who work in women-predominant specialties are often paid less,^2,19^ our simulations may not fully characterize the association of gender inequities with wealth for these subgroups. However, review of unadjusted average salaries of non-MD faculty suggests a decrease in salary gaps over time that parallels implementation of GEI. In the pre-GEI era, unadjusted percentage differences in A+B salary for women vs men were −9.2%, −6.7%, and −7.5% for assistant professor, associate professor, and professor non-MD faculty, respectively. By 2016, these gaps changed to −3.8%, −7.5%, and −6.4%, respectively.

Additionally, it is noted that available data were not stratified by race/ethnicity. Women of all major underrepresented minority groups in the United States earn less than white women, and women in general earn less than men of the same ethnic or racial group.^20^ Thus, the association of gender-based disparities with wealth might be compounded by race/ethnicity.^21^ This remains an underreported subject, and future studies must be aimed at understanding the added difficulties faced by women in science who are also members of underrepresented minority groups.

Although we have demonstrated that the effects of workplace gender disparities can persist past retirement, our analysis assumed that the representative male and female faculty began their careers equally without debt. In the United States, the median total debt among medical school graduates was $185 000 in 2015.^22^ Because repayment of educational debt generally occurs during the first 10 to 20 years of employment, this coincides with the time of greatest disparity between the salaries of women and men. Thus, a female faculty with existing debt may find herself less likely than her male counterpart to make maximum retirement contributions or other investments during repayment. As demonstrated in our simulations, these early gender-based differences could be expected to magnify with time. Again, the association may be even greater for women in underrepresented minority groups, whose educational debt is often higher than that of their white counterparts.^22^ Because educational debt is associated with lower job satisfaction^23^ and physician burnout,^24,25^ it may contribute to attrition rates of women in science.

## Conclusions

We have demonstrated the association of a program to support women faculty with substantially narrowed gender-based salary and TTP differences. Future challenges include maintaining momentum to address small residual gaps and extending research to include other components affecting wealth such as baseline debt and differences by race and ethnicity.

## Notes

Supplement. **eFigure.** Graduated Annuity Estimates Used for Retirement and Salary-Based Investments
