# Supplementary material for: Association of a Simulated Institutional Gender Equity Initiative With Gender-Based Disparities in Medical School Faculty Salaries and Promotions
Source: JAMA Netw Open. 2018 Dec 21;1(8):e186054. doi: 10.1001/jamanetworkopen.2018.6054 (PMC6324345; doi:10.1001/jamanetworkopen.2018.6054)
Supplement: Supplement. — eFigure. Graduated Annuity Estimates Used for Retirement and Salary-Based Investments [file jamanetwopen-1-e186054-s001.pdf]

Supplementary Online Content

Rao AD, Nicholas SE, Kachniarz B, et al. Association of a simulated institutional gender equity initiative with gender-based disparities in medical school faculty salaries and promotions. *JAMA Netw Open*. 2018;1(8):e186054. doi:10.1001/jamanetworkopen.2018.6054

eFigure. Graduated Annuity Estimates Used for Retirement and Salary-Based Investments

This supplementary material has been provided by the authors to give readers additional information about their work.

© 2018 Rao AD et al. *JAMA Network Open.*

eFigure. Graduated annuity estimates used for retirement and salary-based investments.

The retirement and salary-based investment portfolio was modeled as having a 90/10 equity/bond composition at the start of the career of each representative faculty member with a goal 20/80 equity/bond composition at time of retirement. The simulation assumed a linear transition of the investment portfolio composition starting 25 years before retirement and was identical for all simulated scenarios reported in this study.

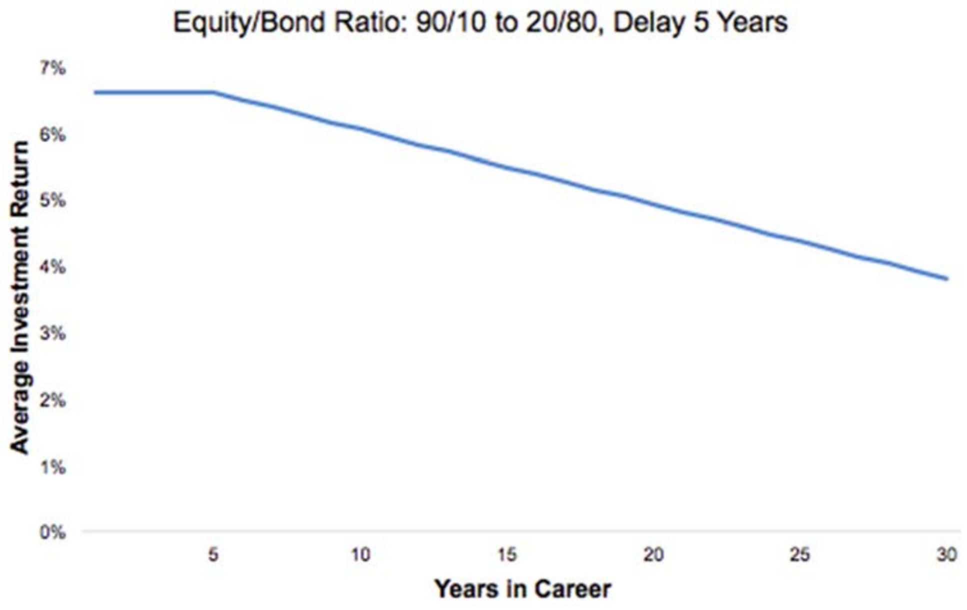

© 2018 Rao AD et al. *JAMA Network Open.*
